# Proton‐beam therapy: are physicists ignoring clinical realities?

**DOI:** 10.1120/jacmp.v16i3.5710

**Published:** 2015-05-08

**Authors:** Michael D. Mills, R. J. Schulz

**Affiliations:** ^1^ Department of Therapeutic Radiology Yale University

The timely guest editorial this month is authored by the distinguished R. J. Schulz, Ph.D. from Yale University. As physicists, we must forever resist being blinded by the technology, and force ourselves to ask some critical questions. How much does this cost? How many quality‐adjusted, life‐years are we really buying for our patients? To whom should the bill be sent and why? What standards should be used to determine cost/benefit of new technologies? Medical physicists would do well to consider these questions and the impact of Dr Schulz's arguments.

## Introduction

Twelve years ago M. Goitein^(1)^ and five years ago A. R. Smith^(2)^ presented excellent reviews of the physics of proton‐beam therapy (PBT) at times when the number of such hospital‐based facilities in the US could be counted on one hand. There can be little doubt that these reviews stimulated interest in PBT in the medical community, but especially among physicists who were intrigued by the potential of PBT to enhance the therapeutic ratio. Over the past decade, about a dozen such facilities have come online in the US; however, our European cousins appear reluctant to invest in this very expensive and unproven treatment modality. Why so? Consider that the first PBT treatments were administered in 1954 at the Lawrence Berkeley Laboratory, and over the next 30 years at a number of physics laboratories before the first hospital‐based facilities came online. Approximately 70,000 patients were treated at these provisional facilities without any substantive claims for clinical outcomes in any way superior to those achieved by conventional radiation therapy. Of course, most of these facilities were not equipped to deliver the optimal dose distributions called for by specific disease sites, nor were the available treatment planning systems capable of taking into account the complexities of the human anatomy. These shortcomings are now largely removed with refinements in delivery and treatment planning systems for PBT the equivalent of those for modern X‐ray systems. Also, over this same period, the growth in the number of patients receiving PBT at hospital‐based facilities has grown to the point where the results so achieved warrant comparisons with those for IMRT or SBRT. However, despite these 20 years of hospital‐based experience, to say nothing of those thousands of patients treated at physics laboratories, randomized controlled trials (RCT) to determine the efficacy of PBT remain in short supply.

## Efficacy

Randomized controlled trials (RCT), also referred to as phase III trials, are the recognized gold standards for the determination of the clinical efficacy of one drug or treatment modality compared with another. RCTs are required by the FDA before it will approve a new drug, but not for a new treatment device like a linear accelerator. However, RCTs are expensive, can take years to complete and, depending upon the nature of the disease, may encounter problems associated with patients being reluctant to enter into “blind” studies, or the introduction of improved methods of patient management over the course of the study that it would be unethical to withhold. Single‐arm, phase I studies for toxicity and phase II studies for measures of efficacy provide the vast majority of clinical data upon which the practice of radiation oncology is based. Meta‐analyses are used to determine the efficacy of one drug or treatment modality compared with another for a specific disease site using phase II clinical data obtained from a number of hospitals. Its results are, therefore, retrospective and nonrandomized, but about the best measure of efficacy we have, short of an RCT.

One of the most common measures of therapeutic efficacy is five‐year survival, either “disease‐specific” or “overall”, both measured from the time of diagnosis. “Disease‐specific” is determined from the number of patients who died specifically from their cancers, whereas “overall” is determined from the number of patients who died from all causes. The accuracy of disease‐specific survival depends upon the assessment of the cause of death; a cancer survivor who died from a heart attack may be mistakenly listed as dying from cancer. Overall survival can be misleading in comparative studies when the average age of one cohort differs from that in the other. For example, in comparing surgical resection with radiation therapy for early‐stage lung cancer, radiation is more often used for inoperable, older patients with comorbidities, and surgery for operable, younger, healthier patients. Clearly, the inoperable patients are more likely to die of all causes during the follow‐up period than the operable patients, and their overall survival will be lower for reasons having little to do with how their cancers were treated.

## Tumor cure probability

Dose for dose, there is but a 10% difference between the biological effectiveness of high‐energy X‐rays and protons, and this difference is routinely taken into account in the dose specifications included in PBT clinical reports. Therefore, given the same tumor dose and fractionation schedule, one should anticipate the same level of tumor response for the patient who receives PBT as a corresponding patient who receives SBRT, IMRT or any other X‐ray based modality. This conjecture is borne out by the meta‐analysis of Grutters et al.^(3)^ who discerned the five‐year, disease‐specific survivals for non‐small cell lung cancer (NSCLC) treated by conventional radiation therapy (RT), PBT, carbon ion therapy (CIT), and SBRT, the results of which are presented in Table 1. Note that, although there is significant overlap of the 95% confidence intervals for SBRT and the particle beams, these data clearly show SBRT as no less than the equal of PBT and CIT for NSCLC. These data accepted, then the deciding factors in the choice of an optimum treatment modality for those tumors amenable to radiation therapy devolves to treatment‐related acute and chronic toxicities, radiation‐induced secondary cancers, followed by those of complexity and cost.

**Table 1:** Disease‐specific, five‐year survivals for patients with stage I NSCLC treated by conventional radiotherapy (RT), stereotactic body radiotherapy (SBRT), proton‐beam therapy (PBT), and carbon‐ion therapy (CIT), as obtained by the meta‐analysis of Grutters et al.^(3)^

*RT*	*SBRT*	*PBT*	*CIT*
43(31–56)%	63(50–75)%	52(32–72)%	64(49–80)%

## Dose escalation

The case for PBT rests mainly upon its potential to enhance therapeutic ratios beyond those achievable with any type of X‐ray modality. Therefore, the issue of dose escalation is as critical to PBT as it is for radiation oncology in general. Whether delivering higher doses than those conventionally delivered results in improved clinical outcomes is debatable, and can depend upon the endpoint being evaluated. For example, Kuban et al.^(4)^ in a randomized clinical trial of dose escalation for prostate cancer, 78 Gy versus 70 Gy, found that after 12 years of follow‐up, patients receiving the higher dose had a lower rate of biochemical failure (50% vs. 65%). However, the 12‐year, disease‐specific survivals were essentially the same (95% versus 99%), whereas the 12‐year overall survival of the 70 Gy group was higher than that for the 78 Gy group (69% versus 57%). As pointed out by Schulz and Kagan,^(5)^ similar data were included in a report by Eade et al.^(6)^ in a retrospective study of over 1,500 patients. The role of dose escalation in tumor control, and the optimum doses and dose rates for most cancers, are seemingly under constant review. Clearly, a more detailed discussion is beyond the scope of the present paper. Suffice it to say that the benefits of dose escalation with protons are likely to remain as elusive as they are currently for X‐rays.

## Morbidities

Because estimates of toxicity are highly subjective (with different physicians grading the same patient differently, and different patients experiencing different levels of discomfit for the same degree of toxicity), the uncertainties associated with graded toxicity levels are generally far greater than those for survival. And these uncertainties are only compounded when data from different clinical reports are combined in meta‐analyses.

Lacking supportive clinical evidence, the arguments presented by those who favor PBT are based mainly upon computer‐generated dose distributions that, not surprisingly, show lower doses to organs at risk (OAR) from PBT compared with those from any of the X‐ray–based modalities. Thus, based upon these advantageous dose distributions, PBT facilities routinely treat any of the tumors that would otherwise be treated by high‐energy X‐rays, the rationale being that any sparing of OAR will reduce toxicities and thus improve the patient's performance status and quality of life. Unfortunately, this rationale has yet to be supported by clinical experience. For example, Grutters et al.^(3)^ also compared the incidence of grade 3–4 pneumonitis, dyspnea, and esophagitis following the aforementioned treatment modalities, and their findings are presented in Table 2. Although limited in scope, these data do not suggest any advantage of PBT over SBRT. The reader is reminded that, when dealing with subjective evaluations of low‐incidence morbidities carried out in different clinics, reliable data are hard to come by. Whether the application of PBT to NSCLC will ultimately result in lower levels of toxicity than SBRT remains to be determined and, due to the small differences in toxicity levels, only by an RCT.

**Table 2:** Grade 3/4 treatment morbidities, as obtained by the meta‐analysis of Grutters et al.^(3)^ Incidence and 95% confidence data are rounded to nearest tenth. When the number of events was zero, only the upper limit of confidence was calculated.

	*Events*	*At Risk*	*Incidence*
Pneumonitis			
RT	2	867	0.2(0.0–0.8)%
SBRT	16	800	2.0(1.1–3.2)%
PBT	1	126	0.8(0.0–4.3)%
CIT	3	210	1.4(0.3–4.1)%
Irreversible Dyspnea			
RT	5	980	0.5(0.2–1.2)%
SBRT	6	769	0.8(0.3–1.7)%
PBT	0	58	0.0(6.2)%
CIT	0	210	0.0(1.7)%
Esophagitis			
RT	1	831	0.1(0.0–0.7)%
SBRT	2	840	0.2(0.0–0.9)%
PBT	0	126	0.0(2.9)%
CIT	none reported		

The prostate being one of the prime targets for PBT, one finds numerous phase II and retrospective reports on post‐treatment morbidity. Slater et al.^(7)^ and Mayahara et al.,^(8)^ actual PBT practitioners, report higher rates of GU complications but comparable rates of GI complications, compared with those reported for 3D CRT and IMRT. Of four retrospective studies^9^, ^10^, ^11^, ^12^ that compared toxicities following PBT or IMRT, three showed but minor differences between the two modalities, while the study by Sheets et al.^(12)^ showed significantly higher GU toxicity following PBT. That these findings do not support the expectations gleaned from computed dose distributions may be due to the doses to OAR from IMRT being close to, or just below, toxicity thresholds, or that problems arise between the development of a PBT treatment plan and its execution.

## Treatment uncertainties

Why the levels of toxicity following PBT are seemingly no different from those following IMRT may be due to the difficulties of achieving *in vivo* the dose distributions visualized in computer‐generated treatment plans. Consider that the depth of penetration of a proton beam is directly proportional to its energy, but inversely proportional to the densities of tissues traversed. Therefore, a passively scattered beam's two‐dimensional energy profile must have the energy at every pixel adjusted so that the spread‐out Bragg peak (SOBP) below that pixel conforms to the distal and proximal surfaces of the tumor. If there is bone under pixel #27, then the energy of the beam exiting that pixel must be higher than the energy of a beam passing through pixel #30 that does not encounter bone. For example, in the treatment of a lung tumor, the impact of the ribs and normal lung included in each treatment field requires the construction of first, a range‐shifting filter that provides the highest and lowest energies required at any point in the treatment field, and second, a tissue‐compensating filter that takes account of the thickness and density of tissues under each pixel. Thus, unique range‐shifting and tissue‐compensating filters are required for each field for each patient under treatment.

If the dose distribution actually delivered is to be the same as the one depicted in the treatment plan, each field must be precisely positioned and matched to the patient's anatomy, on a day‐to‐day basis. However, it is at this point that significant uncertainties may be encountered. If, due to patient positioning or subsequent movement, the rib that had been under pixel #27 is now under #30, the SOBP under #27 will overshoot the distal edge of the tumor, and that under #30 will undershoot the proximal edge. Unlike X‐ray beams, portal imaging, to say nothing about real‐time tracking, is not possible for proton beams. There is no way to confirm that the spread‐out Bragg peak for each field conforms to the tumor volume. PBT dose distributions are far more sensitive to setup errors and patient movement than X‐ray beams. To compensate for these uncertainties, the radiation oncologist has little choice but to increase the margins of the planned treatment volume, thus compromising PBT's touted pinpoint accuracy and increasing the probability of irradiating OARs.

## Secondary malignant neoplasms

It has long been recognized that exposure to ionizing radiations may cause what are referred to as secondary malignant neoplasms^*^ (SMN) which may appear at any time between five and forty years postexposure. The incidence of such neoplasms depends upon the dose and dose rate, the nature and volume of the tissues exposed, the age and sex of the subject, and the type of radiation. Constine et al.^(13)^ report that children having received radiation therapy (RT) or chemotherapy (CT) are more prone to developing SMN than adults, and female children are more prone than males. The standardized incidence ratio (the number observed to the number expected) for SMN in children given CT for Hodgkin's disease was only slightly lower than that for those who underwent RT (13.16 vs. 14.20). In a similar vein, Reulen et al.^(14)^ and Basu et al.^(15)^ found that between 5% and 7% of female children who received RT, CT, or RT+CT, went on to develop breast cancer.

Next to Hodgkin's disease, the majority of clinical reports on SMN are for children and adults with cranial lesions treated by radiosurgery, and for men with prostate cancer. Following cranial irradiations, Rowe^(16)^ reports but one cranial SMN in 4,877 patients after 29,916 patient‐years of follow‐up, whereas 2.47 would have been expected. In a literature survey and a summary of their own results, Muracciole and Regis^(17)^ conclude that the relative risk of a SMN following radiosurgery is less than 1%. By comparison with Hodgkin's disease, these data suggest that the volume and nature of the irradiated tissue are primary determinants for SMN.

The issue of how many more SMN may occur in men with prostate cancer who receive RT as compared with those who are treated by other means was subject to a rigorous evaluation by Berrington de Gonzalez et al.^(18)^ who used SEER data for a 30‐year period, starting in 1973. These data include 76,363 men who received RT as compared with 123,800 who were treated by other means. After adjusting the RT cohort for SMN that would have developed had they not been irradiated, Berrington de Gonzalez and colleagues report 5,548 SMN in the RT cohort (7.3%) versus 8,023 SMN in the treated by other means cohort (6.5%). After adjusting for patient demographic factors such as attained age, year of diagnosis, and clinical stage, the authors report a RR of 1.26 for developing an SMN in those who had RT for prostate cancer compared with those who were treated by other means.

With studies such as that by Berrington de Gonzalez et al., the issue of SMN following X‐ray therapy is being put onto firm ground. Whether the incidence of SMN would be decreased, or perhaps even increased, by the replacement of IMRT by PBT is yet to be determined and, for various reasons, may never be determined. Clearly, the integral dose that results from PBT is lower than that from IMRT. However, as suggested by Gray^(19)^ that lower doses may cause more mutations than higher doses that kill, and by Hall^(20)^ that the neutron contamination in passively scattered proton beams may override the leakage and scatter of X‐rays from IMRT, it is conceivable that PBT would result in more SMN than IMRT. As pointed out by Muller et al.,^(21)^ “Only very large prospective studies which are designed to minimize the influence of possible cofounders will be able to address the real risk of prostate irradiation‐related cancer induction. The available data are clearly not valid nor helpful for guiding any treatment decision.”

## Economics

If over the next decade the survival times of those receiving PBT exceed those receiving any other form of external‐beam therapy by, let us say, 10%–20%, then our health‐care system would be obliged to offer it to all who would so benefit. Therefore, it behooves us to do a rough estimate of what this might cost and how long it might take to reach this goal. In the US, there were about 1.7 million new cases of cancer in 2014. Assuming that 50% of all cancer patients receive radiation therapy and that, of these, 30% are candidates for PBT, such facilities would have the potential to treat 250,000 new cases per year. Consider that each PBT treatment room costs about $40 million and that three patients are treated per hour in an 8‐hr day. If each patient receives 20 fractions (some on a hypofractionated schedule), this facility would be capable of treating 300 patients per year. Therefore, to provide PBT for 250,000 patients annually, over 800 proton‐beam facilities would be required at a total installation cost of over $33 billion. On the other hand, if the survival times of patients receiving PBT remain imperceptibly different from those receiving X‐ray therapy, economic forces would ultimately relegate it to our history books.

Even if PBT proves viable, a one‐time allocation of $33 billion for 800 treatment rooms is not going to happen. Consider a more likely scenario: if $2 billion per year became available for the addition of 50 PBT rooms, 16 years would have passed before the last treatment room was built and equipped. However, during this same period the research, development, and deployment of new cancer therapies (e.g., Gleevec for chronic myelogenous leukemia, and Herceptin for HER2 positive breast cancer) will have proceeded apace, each new drug impacting on the number of patients referred to surgical and radiation oncologists. It is inevitable that, whether it be PBT or IMRT, the number of radiation‐therapy treatments will decrease with time.

And of more immediate concern, although grants from nonprofit foundations have provided the funds for equipment and construction of some of the present PBT facilities, it is more likely that newly planned facilities will have to seek funding from state‐issued bonds, pension funds, venture capitalists, and banks. Thus, their business plans will have to include the not‐insignificant costs of amortization and interest on multimillion dollar loans. Also, as PBT requires a larger, more highly trained staff and expensive maintenance programs, these may double the cost of treating the same patient by IMRT or SBRT. To remain financially viable, patient throughput is vital for PBT, leaving its staff little choice but to treat many of the same patients as would have been treated just as well on the linacs that were in place before the PBT machine arrived.

## Summary

It is eminently clear from phase II evaluations of the more common cancers that the clinical outcomes of PBT are no better — nor any worse — than those achieved by IMRT or SBRT. This broad statement is supported by the reviews of Brada et al.,^(22)^ Lodge et al.,^(23)^ Olsen et al.,^(24)^ and Brada et al.^(25)^ that compare the clinical results achieved by PBT for a wide variety of disease sites with those achieved by more conventional means. The conclusions reached by Lodge and Olsen and their colleagues are essentially the same as those of Brada et al.:^(22)^ “An uncontrolled expansion of clinical units offering as yet unproven and expensive proton therapy is unlikely to advance the field of radiation oncology or be of benefit to cancer patients.”

Disease sites that may benefit from PBT include pediatric solid tumors (neuroblastoma, osteosarcoma, Wilms' tumor, rhabdomyosarcoma, retinoblastoma) of which there are about 2,000 new cases per year, and skull‐based tumors such as chordomas and chondrosarcomas, of which there are fewer than 300 new cases per year. For children, the benefit could be a smaller volume of irradiated tissue, a reduced probability of acute and chronic toxicities, and possibly fewer SMNs. For skull‐based tumors in adults, the benefit would be the sparing of critical nerves. However, the complex boney structures surrounding skull‐based tumors make the planning and accurate implementation of PBT very difficult and, as discussed above, there is no way to confirm that the spread‐out Bragg peak as delivered conforms to the planned treatment volume, nor that critical nerves are spared. Clearly, the treatment of skull‐based tumors is difficult to design and administer and, with an overall incidence of 300, they would best be treated at two or three PBT centers specializing in such treatments than at twenty or more centers that would, on average, see fewer than a dozen such tumors per year.

Despite surgical resection with negative margins or radiation ablation of their primary tumors, about nine out of ten cancer patients die from metastatic disease. If metastases are present at the time of diagnosis, even aggressive attempts at local control are unlikely to improve survival, and systemic therapy may be the only remaining option. The longer survival times that have been achieved over the past decades are due to better understanding of disease processes, screening programs that detect premetastatic disease, new drugs and improved chemotherapeutic agents, and the further integration of radiation therapy into overall treatment strategies. New surgical techniques may decrease operative mortality and morbidity, but are unlikely to increase disease‐specific survival. Likewise, more precisely defined dose distributions may lower the doses to OARs and thereby permit more aggressive tumor doses. However, dose escalation has seemingly reached its limits and more imaginative combinations of radiation and less toxic drugs may be the key to radiation therapy's future.

Radiation oncologists and their staffs hold highly respected places in our society and, as such, have a responsibility to ensure that the limited resources available for health care yield the maximum benefits. Proton‐beam therapy facilities are the most expensive medical devices ever employed for the routine delivery of health care. In view of these multimillion dollar levels of expenditure and the publicity that accompanies each new facility, it is not unreasonable for the cancer patient who is prescribed PBT to anticipate an exceptionally favorable outcome. However, patients given PBT fair no better than those given IMRT or SBRT; by suggesting benefits unlikely to be experienced, the faith of these patients in radiation oncology, as well as that of referring physicians and the entire medical community, will be eroded. The continued promotion of a complex treatment modality whose clinical outcomes are no better than those achieved by less‐expensive modalities is neither medically, morally, nor economically justifiable.
